# A Two-in-One Strategy: Target and Nontarget Site Mechanisms Both Play Important Role in IMI-Resistant Weedy Rice

**DOI:** 10.3390/ijms22030982

**Published:** 2021-01-20

**Authors:** Ru-Ann Yean, Masilamany Dilipkumar, Sadequr Rahman, Beng-Kah Song

**Affiliations:** 1 School of Science, Monash University Malaysia, Bandar Sunway 46150, Selangor, Malaysia; ru.yean@monash.edu (R.-A.Y.); sadequr.rahman@monash.edu (S.R.); 2 Rice Research Centre, Malaysian Agricultural Research and Development Institute (MARDI), MARDI Seberang Perai, Kepala Batas 13200, Pulau Pinang, Malaysia; dilip@mardi.gov.my; 3 Tropical Medicine and Biology Multidisciplinary Platform, Monash University Malaysia, Bandar Sunway 46150, Selangor, Malaysia

**Keywords:** clearfield rice, herbicide-resistant weedy rice, target-site resistance, non-target-site resistance, imidazolinone

## Abstract

The introduction of Clearfield technology allows the use of imidazolinone (IMI) herbicides to control weedy rice. Imidazolinone herbicides stop the acetolactate synthase (ALS) enzyme from synthesizing branched-chain amino acids, resulting in the death of the plant. Since the launch of Clearfield technology in Malaysia in 2010, many farmers have replaced traditional cultivars with Clearfield (CL) rice lines (MR220-CL1 and MR220-CL2). This technology was initially effective; however, in recent years, local farmers have reported the reduced efficacy of IMI herbicides in controlling the spread of weedy rice. Under IMI herbicide treatment, in previous weedy rice studies, the target-site resistance (TSR) mechanism of the *ALS* gene has been suggested as a key factor conferring herbicide resistance. In our study, a combination of *ALS* gene sequencing, enzyme colorimetric assay, and a genome-wide association study (GWAS) highlighted that a non-target-site resistance (NTSR) can be an alternative molecular mechanism in IMI-resistant weedy rice. This is supported by a series of evidence, including a weak correlation between single nucleotide polymorphisms (SNPs) within the *ALS* exonic region and ALS enzyme activity. Our findings suggest that the adaptability of weedy rice in Clearfield rice fields can be more complicated than previously found in other rice strains.

## 1. Introduction

Resistance toward herbicides is a specific process enabling plants to survive the application of herbicide treatments. This can be achieved through either “target-site” or “‘non-target-site” resistance. Target-site resistance (TSR) occurs when there is an alteration at the target site, resulting in the structural or biochemical change of the targeted enzyme in the plant. As a result, the herbicide is no longer be able to bind to its site of action, allowing the plant to survive the herbicide treatments. Non-target-site resistance (NTSR) is a mechanism that does not involve changes at the target site but still enables a plant to survive herbicide application. The potential NTSR mechanisms include reduced herbicide uptake, reduced translocation, reduced herbicide activation, enhanced herbicide detoxification, changes in intra- or intercellular compartmentalization, and enhanced repair of herbicide-induced damage [1]. 

Acetolactate synthase (ALS), also referred to as acetohydroxy acid synthase (AHAS), is a key enzyme that catalyzes the first step in the biosynthesis of branched-chain amino acids (BCAAs), including valine (Val), leucine (Leu), and isoleucine (Ile). This enzyme catalyzes the reaction of two pyruvate molecules, forming acetolactate, which is the first intermediate in the valine and leucine pathways, or is involved in the reaction of pyruvate and 2-ketobutyrate, forming acetohydroxybutyrate, the second intermediate in the isoleucine pathway. The ALS enzyme is the common target for five structurally diverse herbicide groups: sulfonylureas (SU), triazolopyrimidines (TP), pyrimidinylthio-benzoates (PTB), sulfonylamino-carbonyl-triazolinones (SCT), and imidazolinone (IMI), collectively called ALS-inhibiting herbicides [2]. These ALS-inhibiting herbicides have been widely used for controlling weeds worldwide since the 1980s due to their high potency to target plants, allowing a low application rate. Inhibiting this enzyme will affect the synthesis of the BCAAs resulting in plant death [3]. They are also responsible for reducing the amount of herbicides applied to crops. In addition, ALS-inhibiting herbicides are nontoxic to animals, highly selective to plants, and have a broad spectrum of weed control. 

Weedy rice (*Oryza* spp.) is a self-pollinated weed widely distributed in rice fields. It belongs to the same genus as cultivated rice, sharing morphological, physiological, and biochemical characteristics. The weed competes with cultivated rice for water, sunlight, and nutrients, causing yield loss of the cultivated rice. Effective control of the weed relies heavily on imidazolinone (IMI), an ALS-inhibiting herbicide, which was introduced together with cultivated Clearfield (CL) rice under the Clearfield Production System (CPS) [4]. In this system, the *ALS* gene in the CL cultivar was intentionally mutated to tolerate IMI herbicides so that these herbicides could be used to selectively kill weedy rice. Although IMI herbicides are highly effective in controlling weedy rice, a large number of the weedy rice population has developed resistance to IMI herbicides. In Latin America, Europe, and Asia, studies have reported that mutations found in resistant weedy rice resemble the *ALS* mutation in CL rice, thus indicating gene flow is the predominant resistance mechanism that confers herbicide resistance in weedy rice. To date, mutations occurring within the *ALS* locus (Ala122, Pro197, Ala205, Asp376, Arg377, Trp574, Ser653, and Gly654) are commonly known to confer resistance to ALS-inhibiting herbicides in cultivated, wild, and weedy rice [5,6]. For instance, the *ALS* mutation in US IMI-resistant weedy rice was located at Ser653 [7], similar to findings reported by Shivrain et. al. [8], Singh et al. [9], and Rosco et al. [10].

In recent years, the reduced efficacy of IMI herbicides to control the spread of weedy rice has been reported in Malaysia, compromising the yield of cultivated rice [11]. In 2018, it was reported that the average yields of Malaysian CL rice dropped from nine tons per hectare to less than six tons per hectare [12]. Engku et al. [13] demonstrated that weedy rice grown in close proximity to CL rice exhibited a certain level of tolerance to imazapic and imazapyr, which are two categories of IMI herbicides [13]. The widespread occurrence of resistant weedy rice resulting from long-term IMI application on CL cultivars thus warrants investigation of the *ALS* gene in the Malaysian CL and weedy rice populations. 

The objectives of this study were to characterize mutations in the *ALS* gene of the CL cultivars used in Malaysia and determine the molecular mechanism of resistance that confers IMI herbicide resistance in weedy rice. These results are important for a better understanding of the emergence and evolution of the IMI herbicide resistance trait in Malaysian weedy rice and developing a strong basis to address the invasiveness of herbicide-resistant weedy rice in Malaysian Clearfield technology. 

## 2. Results

### 2.1. ALS-Coding Gene Sequence Analysis

Direct Sanger sequencing generated a total of 2086 bp of the aligned *ALS* sequence, which spanned the 1935 bp single exon of the *ALS* gene. Together with four reference sequences comprising *Oryza sativa* ssp. *japonica* (AB049822), *Oryza sativa* ssp. *indica* (CP012610.1), herbicide-resistant weedy rice from the US (AY885675), and CL2 cultivar (this study), 76 weedy rice samples were analyzed, of which 58 and 18 were resistant and susceptible samples. Forty-three SNPs were identified along the exon of the *ALS* gene (Supplementary Table S1). Five out of the 43 SNPs were found useful for differentiating the majority of the susceptible and resistant rice accessions. A single-point mutation at Position 1880 within the exonic region was identified as the functional mutation, associated with the difference between susceptible and resistant weedy rice (except 12 of the IMI-resistant weeds; Table 1 and Table 2). Consistent with previous studies [7,8], the IMI-R weedy rice strains carried the “A” allele whereas the “G” allele was found in IMI-S weedy rice. Such a target-site resistance (TSR) mechanism has been commonly found in other crops, such as Palmer amaranth [14] and flixweed [15]. Surprisingly, 12 IMI-R weedy rice (named IMI-Ra) samples were shown to possess the “G” allele in their *ALS* exonic region (Table 1). It is possible that their IMI resistance trait is controlled by other molecular mechanisms.

### 2.2. Functional Mutation Analysis 

**Figure 1:**
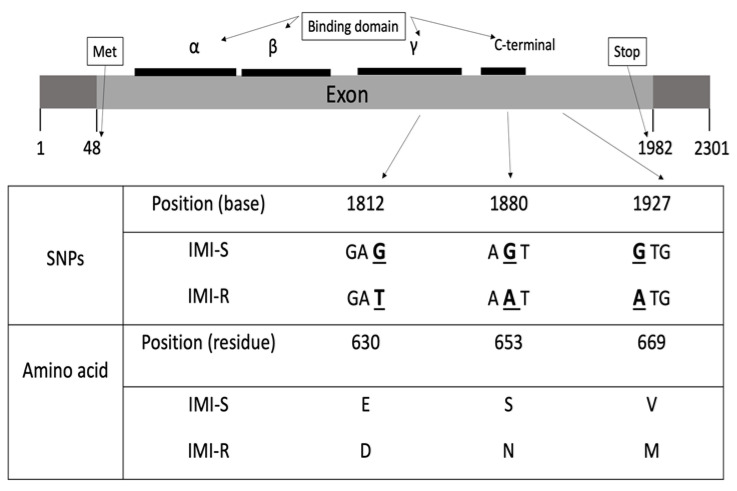
Positions of the three nonsynonymous mutations within the *ALS* gene and the corresponding amino acid listed below the SNPs. The SNPs are underlined and bolded.

In order to validate whether the 43 SNPs would result in amino acid changes that would affect the functions of the ALS enzyme, functional mutation analysis was performed. All rice ALS amino acid sequences were inferred from nucleotide sequences by EMBOSS Transeq (https://www.ebi.ac.uk/Tools/st/emboss_transeq/) and aligned with published *Arabidopsis* amino acid sequence (GenBank ID NM114714) by ClustalW (https://www.ebi.ac.uk/Tools/msa/clustalo/) (Supplementary Figure S1a–c). The 43 exon-derived SNPs are mainly caused by intraspecific variation [16], except for three SNPs located at Positions 1812, 1880, and 1927 (Figure 1). These point mutations resulted in amino acid substitutions from glutamic acid to aspartic acid (Glu^630^Asp), serine to asparagine (Ser^653^Asn), and valine to methionine (Val^669^Met), respectively. These three amino acid alterations were consistent in all IMI-R individuals except for the 12 IMI-Ra rice, which showed no mutation in the *ALS*-coding region.

To study whether these three nonsynonymous mutations are functional, the location of the herbicide-binding domain in the *ALS* gene was determined. In the CL system, *ALS* inhibitory herbicides bind at the herbicide-binding domain in the ALS enzyme. Resistance mutations in the herbicide-binding domain do not change the ALS substrate’s affinity; instead, they decrease the sensitivity of ALS toward the herbicide [17]. Previous studies in *Arabidopsis thaliana* discovered that the ALS enzyme has three herbicide-binding domains spanning the amino acid residues 86–280, 281–451, and 463-639. There is also a C-terminal region comprising residues 646–668, which is important during catalytic activity [18]. Among the abovementioned three nonsynonymous mutations, Glu^630^Asp and Ser^653^Asn are located within the herbicide-binding domain, whereas Val^669^Met occurred outside the binding domain (Figure 1). The mutation at Ser^653^ has also been suggested as the key mutation conferring IMI resistance in the US IMI herbicide-resistant weedy rice [7,8]. The nonsynonymous mutation Glu^630^Asp does not account for IMI resistance in this study because the residue was found different between the susceptible cultivar *Oryza sativa* spp. *indica* (Asp^630^) and *japonica* (Glu^630^).

### 2.3. Haplotype Analysis

**Figure 2:**
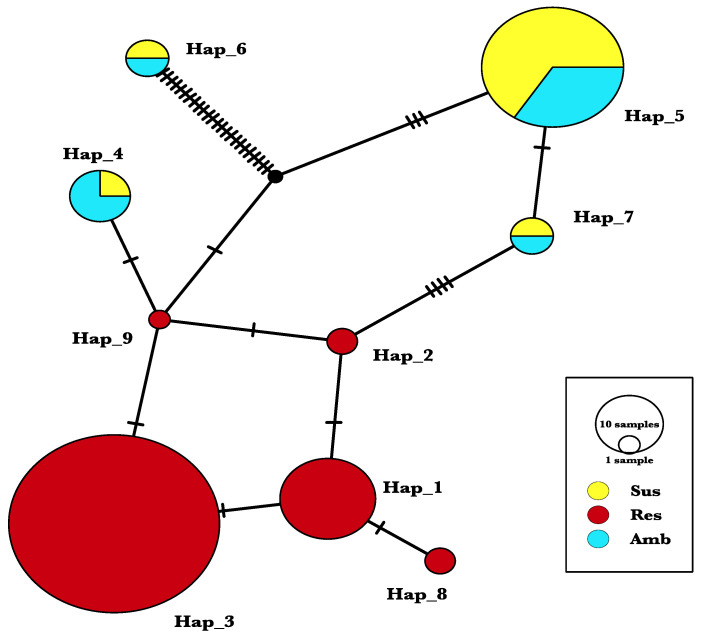
Haplotype analysis of the *ALS* gene. All weedy rice samples are grouped into nine haplotype groups. Sus: IMI-susceptible weedy rice; Res: IMI-resistant weedy rice; Amb: IMI-R ambiguous weedy rice.

Figure 2 shows the result of weedy rice samples clustering together based on their *ALS* gene sequence similarity. Together with the CL rice, 46 of the IMI-R weedy rice are clustered under the haplotype group Hap_3, with two point mutations differentiating the samples from the other IMI-R samples clustered under the Hap_1, Hap_2, Hap_8, and Hap_9. The IMI-S weedy rice can be found in Hap_4, Hap_5, and Hap_7, clustering with the IMI-Ra weedy rice, showing closer genotypic characteristics to the IMI-S samples and possessing no *ALS* functional mutation. Wild rice under Hap_6 has a significant difference in their genotypic characteristics from the weedy rice. One IMI-R weedy rice clustering with the wild rice, possibly implicating gene flow between them.

### 2.4. Enzyme Colorimetric Assay

**Figure 3:**
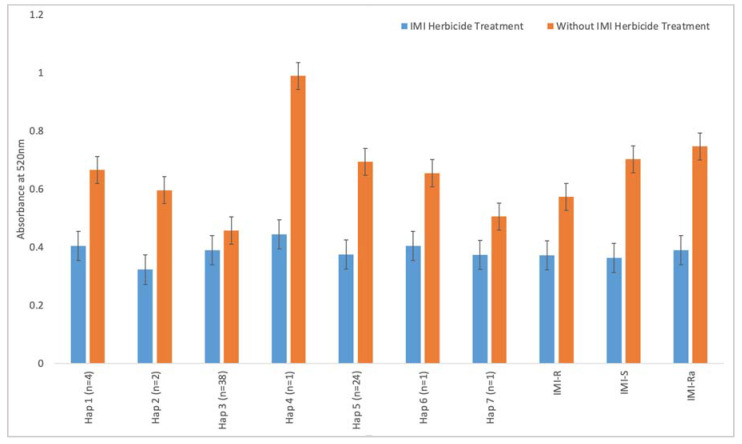
ALS enzyme colorimetric assay, with bar plots showing the absorbance reading of weedy rice samples at 520 nm. The letter “n” represents the number of samples in each haplotype group. IMI-R, IMI-S, and IMI-Ra represent the average absorbance reading of resistant, susceptible, and ambiguous samples, respectively.

IMI-R weedy rice has a lower absorbance reading compared to IMI-S weedy rice when they are not treated with the IMI herbicide (Figure 3). Similarly, under the condition of no IMI treatment, the 12 IMI-Ra samples show a higher absorbance reading compared with IMI-R samples. To examine the correlation between the weedy rice’s genotype (*ALS* gene) and phenotype (ALS enzymatic activity), Spearman’s correlation test was performed using the nonsynonymous SNPs (nucleotides 1812, 1880, and 1927) and colorimetric enzyme assay data. The result showed a weak positive correlation between the ALS enzyme activity and nonsynonymous mutations (r = 0.062, *p* < 0.05), implicating potential influences contributed by other genes.

### 2.5. Genome-Wide Association Studies (GWAS) 

**Figure 4:**
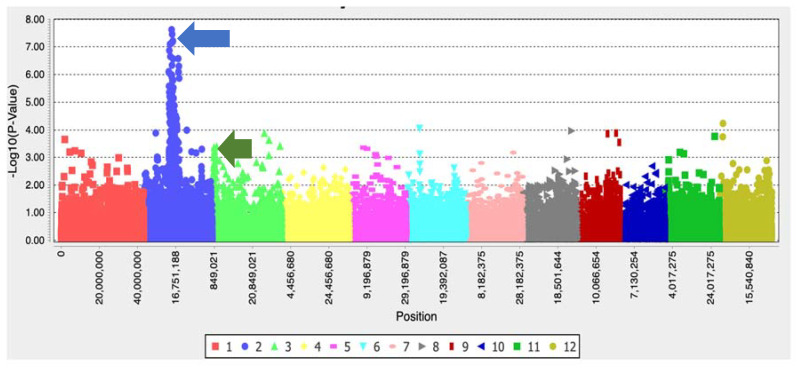
Manhattan plot showing a peak on chromosome 2 (blue arrow) (with –log10 (*p*-value) > 5.8) where the *ALS* gene is located. A lower peak on chromosome 3 (green arrow) was identified as a potential gene contributing toward IMI resistance in weedy rice.

The *p*-value significance threshold on the Manhattan plot was calculated based on the Bonferroni-corrected threshold with an alpha value of 0.05 using the equation *p*-value/total number of SNPs under study. The *p*-value significance threshold after –log10 transformation is 5.8. One significant peak was observed on the chromosome 2 (Figure 4). The BLAST analysis results (Supplementary Figure S2) showed that the peak harbors a 100% identity acetolactate synthase (*ALS*) gene of *Oryza sativa* (GenBank ID ADR72641.1).

## 3. Discussion

### 3.1. Functional Mutation of the ALS Gene and Gene Flow from Clearfield to Weedy Rice 

A single-point mutation at Position 1880 does not fully explain the difference between susceptible and resistant weedy rice (Table 1). Although 46 out of 58 of the IMI herbicide-resistant weedy rice strains (named IMI-R) carry the “A” allele, which gives rise to an amino acid asparagine (Asn) residue instead of serine (Ser) in the “G” allele, the “G” allele was also found in 12 IMI-Ra weedy rice samples. Most of the 43 exon-derived SNPs are due to intraspecific variation, and only five SNPs are consistently found differentiating between the resistant and susceptible weedy rice (Table 2). Unlike the Ser^653^Asn mutation, we found another nonsynonymous mutation, Glu^630^Asp, that could not differentiate between the resistant and susceptible rice. The reason Glu^630^Asp is not a functional mutation for IMI resistance in this study can be explained by the structural orientation of the herbicide binding to ALS. Given that structurally different ALS-inhibiting herbicides orientate differently in the herbicide-binding domain, amino acid substitution within the binding domain can confer resistance to some, but not other, ALS-inhibiting herbicides [18,19].

From the haplotype network diagram (Figure 2), 46 of the IMI-R weedy rice are grouped together with CL in Hap_3, indicating a gene flow from the CL cultivars to the weeds. Three IMI-R samples, each located in Hap_2, Hap_8, and Hap_9, clustering neither with CL rice nor IMI-R rice. This can be explained by the heterozygosity variation in their *ALS* gene. However, this intraspecific variation does not affect the resistance trait in the IMI-R weedy rice. IMI-S wild rice (*Oryza rufipogon*) was found clustered with an IMI-R weedy rice sample (RDA6) in Hap_6 and is genetically distant from the other weedy rice samples. The IMI-R weedy rice that clustered with wild rice in Hap_6 is believed to have acquired a wild rice allele through gene flow. Moreover, samples clustered in Hap_4, Hap_5, and Hap_7 have a combination of memberships with IMI-Ra and IMI-S; more remarkably, a total of 12 IMI-Ra weedy rice are found clustered with IMI-S weedy rice. This finding is consistent with the results of the functional mutation analysis, which revealed that 12 IMI-Ra weedy rice carried no functional mutation at Position 1880 of the nucleotide. This suggests that the functional mutation Ser^653^Asn is not the only factor contributing to the resistance mechanism in weedy rice.

### 3.2. ALS Enzyme Activity 

From the results shown in Figure 3, the absorbance readings of each weedy rice sample were tabulated based on their haplotype grouping in order to compare the functionality of the ALS enzyme. Under the condition without IMI herbicide treatment, Hap_1, Hap_2, and Hap_3, consisting of IMI-R weedy rice, showed a significantly lower absorbance reading (*p* < 0.001, Student’s *t*-test) compared to IMI-S samples (carrying genotypes of Hap_4, Hap_5, Hap_6, and Hap_7). The average absorbance readings for these groups of IMI-R weedy rice with and without IMI herbicide treatment are 0.3726 and 0.5729, respectively, whereas the average readings for Hap_4, Hap_5, Hap_6, and Hap_7 are 0.3978 and 0.7968 with and without IMI herbicide treatments. There is no significant difference observed between IMI-R and IMI-S groups treated with the IMI herbicide (*p* > 0.05, Student’s *t*-test; Figure 3). This is in line with the weak positive correlation (r = 0.062, *p* < 0.05) between the ALS enzyme activity and the three nonsynonymous mutations. Two additional Spearman correlation tests on individual SNP at the position 1880 and all 43 SNPs within the *ALS* gene showed a weak negative correlation, with the r coefficients being −0.095 and −0.083, respectively (*p* < 0.05). Together with the fact that resistant IMI-Ra samples carry the “G” allele, we hypothesized that ALS enzyme activity under IMI herbicide treatment might be influenced by genetic factors other than the *ALS* gene.

### 3.3. Ambiguous Resistance of IMI-Ra 

There are 12 IMI herbicide-resistant weedy rice named IMI-R ambiguous (IMI-Ra) possessing no functional mutation in the *ALS* exonic region. The results of the haplotype analysis revealed that these IMI-Ra weedy rice cluster together with IMI-S weedy rice (Hap_4, Hap_5, and Hap_7), and the results of the ALS enzyme activity assay revealed a similar pattern between IMI-Ra and IMI-S weedy rice, suggesting that these 12 IMI-Ra weedy rice carry an *ALS* gene with characteristics similar to IMI-S weedy rice with the ability to tolerate IMI herbicide treatment. This further supports our hypothesis that the IMI resistance mechanisms in these IMI-Ra weeds are monitored and controlled by genetic processes located outside the *ALS* region. IMI-Ra weedy rice carrying the nonresistance “G” allele at Position 1880 of the nucleotide might have undergone different evolutionary trajectories for achieving its IMI resistance trait. A non-target-site resistance (NTSR) mechanism may possibly explain this inconsistency [20,21]. The lower peak of the Manhattan plot, located at coordinate 849,021 on chromosome 3 (Figure 4), suggests that there is possibly a second gene playing a role in the weedy rice resistance mechanism. Although this gene does not show a significant *p*-value with the MLM model, there is a similar finding with the general linear model (GLM), and a significant peak at the same coordinate on chromosome 3 was observed (unpublished data). This suggests that the MLM model may overcompensate for population structure and kinship, leading to false-negative results. The MLM model is a general approach in GWAS studies to control false positives and is also able to induce false negatives due to overfitting of the model where some potential associations can be missed [22]. Furthermore, the lower peak on chromosome 3 shares a 99.75% similarity with *japonica* AAA-ATPase (GenBank ID XM_015772919.1), which is related to the coenzyme transport and metabolism functions. These findings are generally consistent with the weak positive correlation between phenotypic classes of ALS enzyme activity and the frequency of the mutant alleles (Table 3) and the observation of the nonresistant “G” allele of the *ALS* gene in the IMI-Ra accessions. Although *ALS* may not be the sole key player in IMI resistance, other genes, such as AAA-ATPase, might influence IMI herbicide resistance among the weeds through NTSR mechanisms.

Among the NTSR mechanisms is the reduction in herbicide translocation. According to a previous study on the role of translocation in glyphosate herbicide resistance, a modification of the transporter causing reduced translocation explains the glyphosate resistance in weeds [23]. Delye et al. [1] suggested that NTSR is most likely the major type of resistance of grass weeds to ALS inhibitors, albeit rarely reported in weeds. A recent study has reported that individuals of poppy (*Papaver rhoeas*) with identical genotypes to *ALS* exhibit different herbicide-resistant phenotypes. Given that these differences were attributed to the NTSR mechanism, coupled with a report reiterating the importance of NTSR to ALS inhibitors [24], it appears likely that the same, or similar, NTSR mechanisms contribute to IMI resistance in the weeds of the present study. The mechanism of IMI uptake by the plant, movement of IMI herbicide, and how the ATPase transporter plays a role in this mechanism remains unknown. A limitation in the present GWAS analysis affecting the significant *p*-value could be due to the smaller number of SNPs used, possibly causing a false-negative result. Further work, such as gene expression assessments, will provide more insights on the identification of potential genes that have contributed to or play a role in IMI herbicide resistance mechanisms in weedy rice. This would potentially help in the management of IMI-resistant weedy rice and provide a resolution for the reduced yields in the CL rice fields.

## 4. Materials and Methods

### 4.1. Plant Materials 

Seeds of weedy rice were collected from CL rice fields across seven states in peninsular Malaysia, including Pahang, Penang, Kelantan, Perak, Terengganu, Kedah, and Selangor, following a zig-zag pattern. The samples were sown in pots, and herbicide screening was performed in the glasshouse of the Malaysian Agricultural Research and Development Institute (MARDI), Seberang Perai. Five days after sowing, each pot with 10 seeds from every weedy rice sample was treated with registered IMI herbicides (OnDuty^®^, BASF (M) Pvt. Ltd., Selangor, Malaysia) at 300 g ai/ha, whereas the control pot was treated with water. After 14 days, the surviving and dying plants were counted, considered as IMI-resistant (IMI-R) and IMI-susceptible (IMI-S) biotypes, respectively. A total of 58 IMI-resistant and 18 IMI-susceptible weedy rice were sorted from 62 CL rice fields across the seven states (Supplementary Table S2). Seeds were incubated in a 32 °C incubator for shoot and root germination. After 3–4 days, the germinated seeds were sown into a pot filled with potting soil and fertilized once every 2 weeks. CL rice MR220CL2 was used as the resistant rice control. 

### 4.2. Genomic DNA Samples

Leaves of one-month-old seedlings (at the 5-leaf stage) were harvested for genomic DNA extraction. Approximately 150 mg of young leaves were collected and directly placed in liquid nitrogen. The frozen leaves were ground into a fine powder. Total genomic DNA was extracted using a QIAGEN DNeasy Extraction kit (QIAGEN, Valencia, CA). The DNA samples were quantified on 0.8% agarose gel and a fluorometer (Qubit 4 Fluorometer, Thermo Fisher), followed by dilution in 30 ng/µL of deionized water. 

### 4.3. Gene Amplification and Sequence Analysis

A full-length *ALS* gene (2301 base pair) was amplified by polymerase chain reaction (PCR) using the two sets of primers designed from the *ALS* sequence of *Oryza sativa* (AB049822). The primer pairs, along with the annealing temperatures, are listed in Supplementary Table S3. Each 25 µL PCR reaction mix contained 30 ng of genomic DNA, 10 µM of each primer, 1 U of Taq polymerase (GoTaq, Promega, Madison, WI, USA), 11.5 µM of MgCl_2_, 1 unit of buffer solution, and 0.5 µM of dNTPs. The PCR mixture was then topped up with nuclease-free water to a total volume of 25 µL. The reactions were loaded in a MyCycler (BioRad MyCycler Thermal Cycler, 115V) thermal cycler for the following temperature profiles: one cycle at 98 °C (3 min); 35 cycles at 98 °C (30 s), annealing temperature (45 s), and 72 °C (1 min 30 s); and a final cycle at 72 °C (5 min). All amplified products were visualized in 1% agarose gel for quality checking before Sanger sequencing by an outsource company (1st Base Sdn. Bhd). Sequence contigs were aligned and checked for *ALS* mutations using the Sequencher 4.10.1 software (Gene Codes Corp., Ann Arbor, MI, USA). Amino acid sequences were generated using the EMBOSS Transeq database (https://www.ebi.ac.uk/Tools/st/emboss_transeq/) and aligned by ClustalW to identify mutations.

### 4.4. Enzyme Extraction 

Soluble ALS proteins were extracted according to Singh et al. [25], with minor modifications. Briefly, 500 mg of fresh leaf tissue (5-leaf stage) was collected and directly placed in liquid nitrogen. The frozen leaves were ground into a fine powder and added into 5 mL of protein extraction buffer consisting of 100 mM of potassium phosphate buffer (pH 7.5), 10 mM of sodium pyruvate, 5 mM MgCl_2,_ 5 mM EDTA, 100 µM of Flavin adenine dinucleotide (FAD), and 10% glycerol. The mixture was centrifuged at 4 °C for 20 min at 22,000× *g*. The supernatant was transferred to a new tube and mixed with an equal volume of saturated ammonium sulfate. The mixture was incubated in ice for 30 min and centrifuged at 4 °C for 20 min at 22,000× *g*. The supernatant was discarded, and the pellet containing the protein was resuspended in 700 µL of the buffer solution containing 50 mM potassium phosphate buffer (pH 7.5), 100 mM sodium pyruvate, 10 mM MgCl_2,_ 1 mM EDTA, 10 µM of Flavin adenine dinucleotide (FAD), 100 mM NaCl, and 1 mM thiamine pyrophosphate (TPP).

### 4.5. Colorimetric Enzyme Activity Assay

Enzyme activity was tested using IMI herbicide. Fifty-two microliters of the enzyme (in extraction buffer) was added to an equal volume of 70% of IMI herbicide (OnDuty^®^, BASF (M) Pvt. Ltd., Selangor, Malaysia). The mixture was incubated at 37 °C for 1 h to facilitate acetolactate production. The reaction was stopped by adding 21 µL of 5% sulfuric acid to the mixture, followed by incubation at 60 °C for 15 min. After incubation, tubes were spiked with 175 µL of color-changing solution containing 0.32 g of NaOH, 0.12 g of 1-naphthol, and 0.01 g of creatine in 4 mL of deionized water and reincubated at 60 °C for 15 min. After incubation, 200 µL of the reaction mixture was added to a 96-well plate to determine the enzyme activity by studying the color change using a microplate reader spectrophotometer (Infinite 200 Pro, TECAN) at a 520 nm wavelength.

### 4.6. Genotyping-by-Sequencing (GBS) and Genome-Wide Association Studies (GWAS)

Extracted DNA samples were digested for 3 h at 75 °C with the restriction enzyme *Ape*KI (New England Biolabs, Ipswich, MA, U.S.A), and genotyping-by-sequencing (GBS) was performed based on the methods outlined by Vigueira et al. [26] using the Illumina Hiseq 2000 platform (Illumina Inc., San Diego, CA). Raw sequence reads in a FASTQ format were processed using a standard TASSEL-GBS pipeline version 5.0 (http://www.maizegenetics.net/) [27]. Briefly, filtered reads were aligned to the rice genome MSU 6.0 (http://rice.plantbiology.msu.edu) using the Burrows–Wheeler alignment (BWA) tool [28]. This allowed a maximum of four mismatches and no gap within 5 bp at the end of each read. Loci with more than 30% of missing data and monomorphic data were discarded. Individuals with more than 95% of missing data were removed and not included in this study. After filtering, a total of 32,053 SNPs were retained for GWAS analysis. The mixed linear model (MLM) in the TASSEL-GBS program was used to generate a Manhattan plot for association analysis.

## Figures and Tables

**Table 1:** Comparison of nucleotide sequences at Position 1880 of the *ALS* gene between IMI-R, IMI-Ra, IMI-S weedy rice, and CL cultivar. Resistant rice carries the “A” allele and susceptible rice carries the “G” allele. All IMI-R samples show the “A” allele except for the 12 IMI-Ra, which possess the ‘G” allele. IMI-S: IMI-susceptible weedy rice; IMI-R: IMI-resistant weedy rice; CL rice: Clearfield rice; IMI-Ra: IMI-resistant ambiguous weedy rice.

Nucleotide Sequences at Positions 1879–1881	Rice Accession
AGT	IMI-S weedy rice (n = 18); IMI-Ra weedy rice (n = 12): RDA6, RDA4, RKI2, RSK6, RSK9, RTA, RDA4a, RDA5a, RPA4, RTAa, RDE2a, RPC3
AAT	Clearfield rice CL2; IMI-R weedy rice (n = 46)

**Table 2:** Nucleotide and amino acid changes differentiating the majority of the IMI-R with IMI-S weedy rice.

Nucleotide Position	Nucleotide Change	Domain	Altered Amino Acid	Amino Acid Group Change
1239	GCG-GCA	-	Neutral	-
1754	AGT-AGC	-	Neutral	-
1812	GAT-GAG	Gamma	Asp^630^Glu	-
1880	AGT-AAT	C-terminal	Ser^653^Asn	-
1927	GTG-ATG	-	Val^669^Met	-

**Table 3:** Spearman’s correlation test. A weak positive correlation was observed between the genotypic and phenotypic data. Genotypic data: functional mutations (Positions 1812, 1880, and 1927) in the *ALS* gene. Phenotypic data: enzyme colorimetric assay.

Spearman Correlation Test			
		Phenotype	Genotype
Correlation Coefficient	**Phenotype**	1.000	0.062 *
	**Genotype**	0.062 *	1.000

* Correlation is significant at the 0.05 level (2-tailed).

## Supplementary Materials

The following are available online at https://www.mdpi.com/1422-0067/22/3/982/s1.
