# Supplementary material for: A Two-in-One Strategy: Target and Nontarget Site Mechanisms Both Play Important Role in IMI-Resistant Weedy Rice
Source: Int J Mol Sci. 2021 Jan 20;22(3):982. doi: 10.3390/ijms22030982 (PMC7863736; doi:10.3390/ijms22030982)
Supplement: Supplementary file 1 [file ijms-22-00982-s001.pdf]

SUPPLEMENTARY DOCUMENTS

Supplementary Table S1. Nucleotide differences, along with amino acid changes, in weedy rice samples collected from CL rice field.

Nucleotide position	Nucleotide change	Altered amino acid	Change of amino acid group
9	ACG - ACA	Neutral	-
31	ACC - GCC	Thr to Ala	-
34	TTG - CTG	Neutral	-
47	GCG – GGG	Ala to Gly	-
79	CGA – GGA	Arg to Gly	Charged to polar
91	CTT - TTT	Leu to Phe	-
104	GGC - GCC	Gly to Ala	-
124	GTC – TTC	Glu to Lys	Negative charged to positive
199	CCG – ACG	Gly to Cys	-
711	TGT – CGT	Thr to Ala	-
717	AGT – GGT	Ser to Pro	-
744	GGG – AGG	Pro to Ser	-
753	TGC – CGC	Thr to Ala	-
867	TGA – CGA	Thr to Ala	-

877	CGG - TGG	Ala to Thr	-
894	CGG – TGG	Ala to Thr	-
897	CAT – TAT	Val to Ile	-
945	TGA – CGA	Thr to Arg	Polar to positive charged
948	TCC – CCC	Arg to Gly	Positive charged to polar
1002	GGT – CGT	Pro to Ala	-
1029	ATT - GTT	Stop to Gln	-
1035	CGT – TGT	Ala to Thr	-
1060	AGG – GGG	Phe to Pro	-
1119	GCG – GCA	Neutral	-
1201	GAC – CAC	Leu to Val	-
1203	GAC – CAA	Leu to Val	-
1239	GCA – GCG	Neutral	-
1290	AAG – AAA	Neutral	-
1323	GCT - GCC	Neutral	-
1353	GGG – GGT	Neutral	-
1380	GGA – GGG	Neutral	-
1564	TTG – CTG	Neutral	-
1593	CCG - CCT	Neutral	-

1635	GTT – GTG	Glu to His	Acidic to aromatic
1665	GCA – GCG	Neutral	-
1695	CCA – CCG	Neutral	-
1707	AGT – AGC	Neutral	-
1740	AAA – AAG	Neutral	-
**1812**	**GAT – GAG**	**Asp to Glu**	-
1815	ACC – ACT	Trp to Stop	-
1848	CCA - CCG	Neutral	-
**1880**	**AGT - AAT**	**Ser to Asn**	-
**1927**	**GTG - ATG**	**Val to Met**	-

Supplementary Table S2. Rice samples used for *ALS* gene sequence analysis in this study.

Sample	No. of sample
**Reference for susceptible weedy rice (Ref-)**<br><br>*Oryza sativa japonica* (Genbank ID AB049822)	1
**Reference for susceptible weedy rice (Ref-)**<br><br>*Oryza sativa indica* (Genbank ID CP012610.1)	1
**Reference for IMI-resistant weedy rice (Ref-)**<br><br>US-HR weedy rice (Genbank ID AY885675)	1

**Malaysian IMI-resistant cultivated rice**<br><br>CL2	1
**Malaysian IMI-resistant weedy rice samples**<br><br>RAA1, RAB2, RAC2, RAD1, RAD2, RAD3, RBA1, RBA2, RBB1, RBB2, RBB3, RBC2, RBC3, RCE, RDA5, RDB1, RDC1, RDC2, RDD1, RDD2, RDE1, RKC, RKD, RKE, RKF, RKG, RKH, RKI1, RKJ, RKK, RKL, RPC1, RPC2, RSK2, RSK8, RSK10, RAD1a, RAD3a, RBA2a, RBB1a, RBB2a, RBB3a, RDA6a, RDE1a, RKEa, RKFa, RDA6, RDA4, RKI2, RSK6, RSK9, RTA, RDA4a, RDA5a, RPA4, RTAa, RDE2a, RPC3	58
**Malaysian susceptible weedy rice samples**<br><br>SAB1, SCD, SDA1, SDA2, SDA3, SKA, SKB, STB, STC, STE, SPC1, SPC5, SPA2, SDA1a, STBa, STEa, SPB1, SPC2	18
**Total**	**80**

Supplementary Table S3. Primers used for the amplification of the *ALS* gene in rice.

Primer (F: forward; R: reverse)	Primer sequence	PCR amplicon size (bp)

ALS_F	CCCAAACCCAGAAACCCTCG	
2R	GCACAATCTTGGCCCTGCT	1147
5F_1	AAATTATGCCGTGGATAAGGC	
5R	TTTTGCATAGAAGTACTTTATTCTC	1153
	(Rajguru et al. 2005)	

```
Ref-Indica       ACAAAGAAGAGTGAAGTCCGTGCCGCCATCAAGAAGATGCTCGATACCCCAGGGCCATAC
Ref-Japonica     ACAAAGAAGAGTGAAGTCCGTGCCGCCATCAAGAAGATGCTCGAGACTCCAGGGCCATAC
Ref-US_HR        ACAAAGAAGAGTGAAGTCCGTGCCGCCATCAAGAAGATGCTCGAGACCCCAGGGCCATAC
SKCL2            ACAAAGAAGAGTGAAGTCCGTGCCGCCATCAAGAAGATGCTCGAGACCCCAGGGCCATAC
RBB3a            ACAAAGAAGAGTGAAGTCCGTGCCGCCATCAAGAAGATGCTCGAGACCCCAGGGCCATAC
RPA4             ACAAAGAAGAGTGAAGTCCGTGCCGCCATCAAGAAGATGCTCGAGACCCCAGGGCCATAC
RDB1             ACAAAGAAGAGTGAAGTCCGTGCCGCCATCAAGAAGATGCTCGAGACCCCAGGGCCATAC
RAC2             ACAAAGAAGAGTGAAGTCCGTGCCGCCATCAAGAAGATGCTCGAGACCCCAGGGCCATAC
RBA1             ACAAAGAAGAGTGAAGTCCGTGCCGCCATCAAGAAGATGCTCGAGACCCCAGGGCCATAC
RBA2a            ACAAAGAAGAGTGAAGTCCGTGCCGCCATCAAGAAGATGCTCGAGACCCCAGGGCCATAC
RPB3             ACAAAGAAGAGTGAAGTCCGTGCCGCCATCAAGAAGATGCTCGAGACCCCAGGGCCATAC
RDA6a            ACAAAGAAGAGTGAAGTCCGTGCCGCCATCAAGAAGATGCTCGAGACCCCAGGGCCATAC
RAD3a            ACAAAGAAGAGTGAAGTCCGTGCCGCCATCAAGAAGATGCTCGAGACCCCAGGGCCATAC
RKEa             ACAAAGAAGAGTGAAGTCCGTGCCGCCATCAAGAAGATGCTCGAGACCCCAGGGCCATAC
RKC              ACAAAGAAGAGTGAAGTCCGTGCCGCCATCAAGAAGATGCTCGAGACCCCAGGGCCATAC
RDD2             ACAAAGAAGAGTGAAGTCCGTGCCGCCATCAAGAAGATGCTCGAGACCCCAGGGCCATAC
RBC3             ACAAAGAAGAGTGAAGTCCGTGCCGCCATCAAGAAGATGCTCGAGACCCCAGGGCCATAC
RSK2             ACAAAGAAGAGTGAAGTCCGTGCCGCCATCAAGAAGATGCTCGAGACCCCAGGGCCATAC
RDD1             ACAAAGAAGAGTGAAGTCCGTGCCGCCATCAAGAAGATGCTCGAGACCCCAGGGCCATAC
RCE              ACAAAGAAGAGTGAAGTCCGTGCCGCCATCAAGAAGATGCTCGAGACCCCAGGGCCATAC
RDE1             ACAAAGAAGAGTGAAGTCCGTGCCGCCATCAAGAAGATGCTCGAGACCCCAGGGCCATAC
RDC1             ACAAAGAAGAGTGAAGTCCGTGCCGCCATCAAGAAGATGCTCGAGACCCCAGGGCCATAC
RBB1a            ACAAAGAAGAGTGAAGTCCGTGCCGCCATCAAGAAGATGCTCGAGACCCCAGGGCCATAC
RAD1a            ACAAAGAAGAGTGAAGTCCGTGCCGCCATCAAGAAGATGCTCGAGACCCCAGGGCCATAC
RSK6             ACAAAGAAGAGTGAAGTCCGTGCCGCCATCAAGAAGATGCTCGAGACCCCAGGGCCATAC
RBA2             ACAAAGAAGAGTGAAGTCCGTGCCGCCATCAAGAAGATGCTCGAGACCCCAGGGCCATAC
RDA4a            ACAAAGAAGAGTGAAGTCCGTGCCGCCATCAAGAAGATGCTCGATACCCCAGGGCCATAC
RSK9             ACAAAGAAGAGTGAAGTCCGTGCCGCCATCAAGAAGATGCTCGATACCCCAGGGCCATAC
RTAa             ACAAAGAAGAGTGAAGTCCGTGCCGCCATCAAGAAGATGCTCGATACCCCAGGGCCATAC
RDE2a            ACAAAGAAGAGTGAAGTCCGTGCCGCCATCAAGAAGATGCTCGATACCCCAGGGCCATAC
RKD              ACAAAGAAGAGTGAAGTCCGTGCCGCCATCAAGAAGATGCTCGAGACCCCAGGGCCATAC
RBB2a            ACAAAGAAGAGTGAAGTCCGTGCCGCCATCAAGAAGATGCTCGAGACCCCAGGGCCATAC
RDA6             ACAAAGAAGAGTGAAGTCCGTGCCGCCATCAAGAAGATGCTCGAGACTCCAGGGCCATAC
RAA1             ACAAAGAAGAGTGAAGTCCGTGCCGCCATCAAGAAGATGCTCGAGACCCCAGGGCCATAC
RDA5             ACAAAGAAGAGTGAAGTCCGTGCCGCCATCAAGAAGATGCTCGAGACCCCAGGGCCATAC
RKF              ACAAAGAAGAGTGAAGTCCGTGCCGCCATCAAGAAGATGCTCGAGACCCCAGGGCCATAC
RTA              ACAAAGAAGAGTGAAGTCCGTGCCGCCATCAAGAAGATGCTCGATACCCCAGGGCCATAC
RBB3             ACAAAGAAGAGTGAAGTCCGTGCCGCCATCAAGAAGATGCTCGAGACCCCAGGGCCATAC
RDA5a            ACAAAGAAGAGTGAAGTCCGTGCCGCCATCAAGAAGATGCTCGATACCCCAGGGCCATAC
RSK10            ACAAAGAAGAGTGAAGTCCGTGCCGCCATCAAGAAGATGCTCGAGACCCCAGGGCCATAC
RDC2             ACAAAGAAGAGTGAAGTCCGTGCCGCCATCAAGAAGATGCTCGAGACCCCAGGGCCATAC
RAD2             ACAAAGAAGAGTGAAGTCCGTGCCGCCATCAAGAAGATGCTCGAGACCCCAGGGCCATAC
RAD3             ACAAAGAAGAGTGAAGTCCGTGCCGCCATCAAGAAGATGCTCGAGACCCCAGGGCCATAC
RSK8             ACAAAGAAGAGTGAAGTCCGTGCCGCCATCAAGAAGATGCTCGAGACCCCAGGGCCATAC
RKFa             ACAAAGAAGAGTGAAGTCCGTGCCGCCATCAAGAAGATGCTCGAGACCCCAGGGCCATAC
RAD1             ACAAAGAAGAGTGAAGTCCGTGCCGCCATCAAGAAGATGCTCGAGACCCCAGGGCCATAC
RDE1a            ACAAAGAAGAGTGAAGTCCGTGCCGCCATCAAGAAGATGCTCGAGACCCCAGGGCCATAC
RDA4             ACAAAGAAGAGTGAAGTCCGTGCCGCCATCAAGAAGATGCTCGATACCCCAGGGCCATAC
STEa             ACAAAGAAGAGTGAAGTCCGTGCCGCCATCAAGAAGATGCTCGATACCCCAGGGCCATAC
SDA2a            ACAAAGAAGAGTGAAGTCCGTGCCGCCATCAAGAAGATGCTCGAGACCCCAGGGCCATAC
SDA1a            ACAAAGAAGAGTGAAGTCCGTGCCGCCATCAAGAAGATGCTCGATACCCCAGGGCCATAC
RBC2             ACAAAGAAGAGTGAAGTCCGTGCCGCCATCAAGAAGATGCTCGAGACCCCAGGGCCATAC
RAB2             ACAAAGAAGAGTGAAGTCCGTGCCGCCATCAAGAAGATGCTCGAGACCCCAGGGCCATAC
RKK              ACAAAGAAGAGTGAAGTCCGTGCCGCCATCAAGAAGATGCTCGAGACCCCAGGGCCATAC
```

Supplementary Figure S1a. Sequence alignment of a region of the *ALS* gene. Red box indicates the resistance-conferring *ALS* mutation from G to A at nucleotide position 1812, causing non-synonymous mutation from glutamate (GAG) to aspartate (GAT).

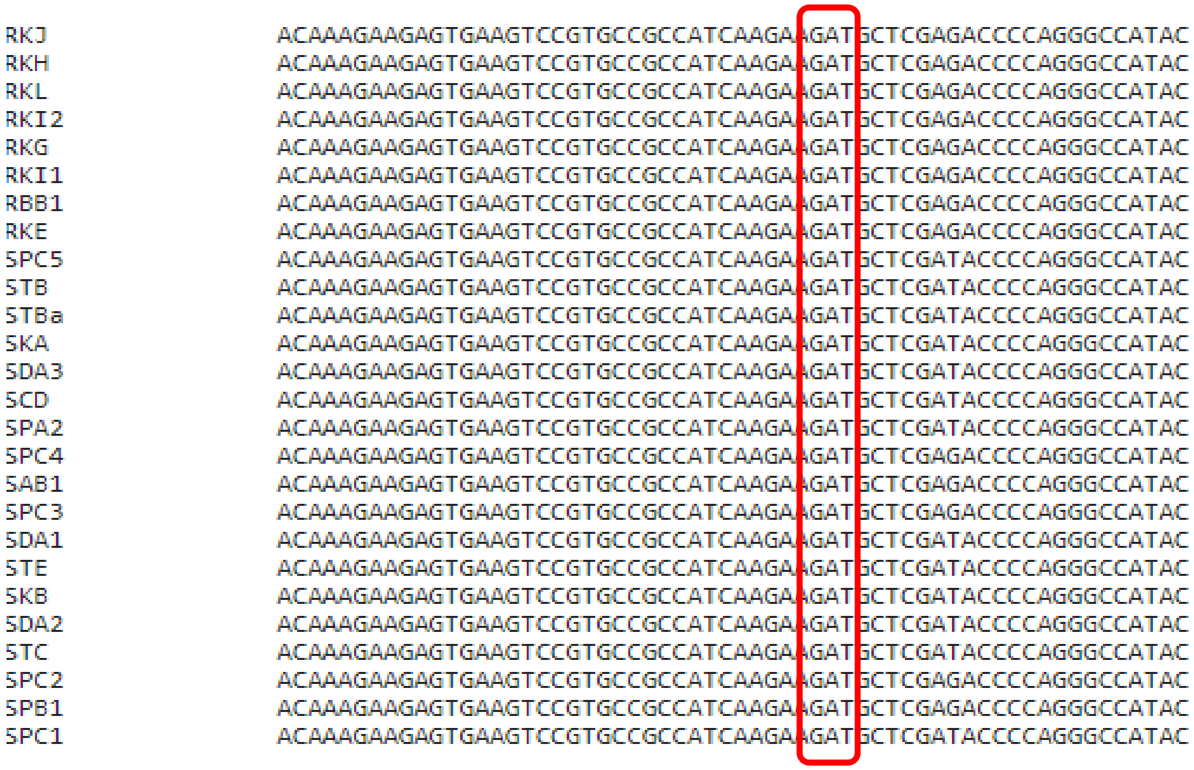

Supplementary Figure S1a. Cont. Sequence alignment of a region of the *ALS* gene. Red box indicates the resistance-conferring *ALS* mutation from G to A at nucleotide position 1812, causing non-synonymous mutation from glutamate (GAG) to aspartate (GAT).

Ref-Indica	TTGTTGGATATCATCGTCCCACACCAGGAGCATGTGCTGCCTATGATCCCAAGTGGGGGC
Ref-Japonica	TTGTTGGATATCATCGTCCCGCACCAGGAGCATGTGCTGCCTATGATCCCAAGTGGGGGC
Ref-US_HR	TTGTTGGATATCATCGTCCCACACCAGGAGCATGTGCTGCCTATGATCCCAAATGGGGGC
SKCL2	TTGTTGGATATCATCGTCCCACACCAGGAGCATGTGCTGCCTATGATCCCAAATGGGGGC
RBB3a	TTGTTGGATATCATCGTCCCACACCAGGAGCATGTGCTGCCTATGATCCCAAATGGGGGC
RPA4	TTGTTGGATATCATCGTCCCACACCAGGAGCATGTGCTGCCTATGATCCCAAGTGGGGGC
RDB1	TTGTTGGATATCATCGTCCCACACCAGGAGCATGTGCTGCCTATGATCCCAAATGGGGGC
RAC2	TTGTTGGATATCATCGTCCCACACCAGGAGCATGTGCTGCCTATGATCCCAAATGGGGGC
RBA1	TTGTTGGATATCATCGTCCCACACCAGGAGCATGTGCTGCCTATGATCCCAAATGGGGGC
RBA2a	TTGTTGGATATCATCGTCCCACACCAGGAGCATGTGCTGCCTATGATCCCAAATGGGGGC
RPB3	TTGTTGGATATCATCGTCCCACACCAGGAGCATGTGCTGCCTATGATCCCAAATGGGGGC
RDA6a	TTGTTGGATATCATCGTCCCACACCAGGAGCATGTGCTGCCTATGATCCCAAATGGGGGC
RAD3a	TTGTTGGATATCATCGTCCCACACCAGGAGCATGTGCTGCCTATGATCCCAAATGGGGGC
RKEa	TTGTTGGATATCATCGTCCCACACCAGGAGCATGTGCTGCCTATGATCCCAAATGGGGGC
RKC	TTGTTGGATATCATCGTCCCACACCAGGAGCATGTGCTGCCTATGATCCCAAATGGGGGC
RDD2	TTGTTGGATATCATCGTCCCACACCAGGAGCATGTGCTGCCTATGATCCCAAATGGGGGC
RBC3	TTGTTGGATATCATCGTCCCACACCAGGAGCATGTGCTGCCTATGATCCCAAATGGGGGC
RSK2	TTGTTGGATATCATCGTCCCACACCAGGAGCATGTGCTGCCTATGATCCCAAATGGGGGC
RDD1	TTGTTGGATATCATCGTCCCACACCAGGAGCATGTGCTGCCTATGATCCCAAATGGGGGC
RCE	TTGTTGGATATCATCGTCCCACACCAGGAGCATGTGCTGCCTATGATCCCAAATGGGGGC
RDE1	TTGTTGGATATCATCGTCCCACACCAGGAGCATGTGCTGCCTATGATCCCAAATGGGGGC
RDC1	TTGTTGGATATCATCGTCCCACACCAGGAGCATGTGCTGCCTATGATCCCAAATGGGGGC
RBB1a	TTGTTGGATATCATCGTCCCACACCAGGAGCATGTGCTGCCTATGATCCCAAATGGGGGC
RAD1a	TTGTTGGATATCATCGTCCCACACCAGGAGCATGTGCTGCCTATGATCCCAAATGGGGGC
RSK6	TTGTTGGATATCATCGTCCCACACCAGGAGCATGTGCTGCCTATGATCCCAAGTGGGGGC
RBA2	TTGTTGGATATCATCGTCCCACACCAGGAGCATGTGCTGCCTATGATCCCAAATGGGGGC
RDA4a	TTGTTGGATATCATCGTCCCACACCAGGAGCATGTGCTGCCTATGATCCCAAGTGGGGGC
RSK9	TTGTTGGATATCATCGTCCCACACCAGGAGCATGTGCTGCCTATGATCCCAAGTGGGGGC
RTAa	TTGTTGGATATCATCGTCCCACACCAGGAGCATGTGCTGCCTATGATCCCAAGTGGGGGC
RDE2a	TTGTTGGATATCATCGTCCCACACCAGGAGCATGTGCTGCCTATGATCCCAAGTGGGGGC
RKD	TTGTTGGATATCATCGTCCCACACCAGGAGCATGTGCTGCCTATGATCCCAAATGGGGGC
RBB2a	TTGTTGGATATCATCGTCCCACACCAGGAGCATGTGCTGCCTATGATCCCAAATGGGGGC
RDA6	TTGTTGGATATCATCGTCCCGCACCAGGAGCATGTGCTGCCTATGATCCCAAGTGGGGGC
RAA1	TTGTTGGATATCATCGTCCCACACCAGGAGCATGTGCTGCCTATGATCCCAAATGGGGGC
RDA5	TTGTTGGATATCATCGTCCCACACCAGGAGCATGTGCTGCCTATGATCCCAAATGGGGGC
RKF	TTGTTGGATATCATCGTCCCACACCAGGAGCATGTGCTGCCTATGATCCCAAATGGGGGC
RTA	TTGTTGGATATCATCGTCCCACACCAGGAGCATGTGCTGCCTATGATCCCAAGTGGGGGC
RBB3	TTGTTGGATATCATCGTCCCACACCAGGAGCATGTGCTGCCTATGATCCCAAATGGGGGC
RDA5a	TTGTTGGATATCATCGTCCCACACCAGGAGCATGTGCTGCCTATGATCCCAAGTGGGGGC
RSK10	TTGTTGGATATCATCGTCCCACACCAGGAGCATGTGCTGCCTATGATCCCAAATGGGGGC
RDC2	TTGTTGGATATCATCGTCCCACACCAGGAGCATGTGCTGCCTATGATCCCAAATGGGGGC
RAD2	TTGTTGGATATCATCGTCCCACACCAGGAGCATGTGCTGCCTATGATCCCAAATGGGGGC
RAD3	TTGTTGGATATCATCGTCCCACACCAGGAGCATGTGCTGCCTATGATCCCAAATGGGGGC
RSK8	TTGTTGGATATCATCGTCCCACACCAGGAGCATGTGCTGCCTATGATCCCAAATGGGGGC
RKFa	TTGTTGGATATCATCGTCCCACACCAGGAGCATGTGCTGCCTATGATCCCAAATGGGGGC
RAD1	TTGTTGGATATCATCGTCCCACACCAGGAGCATGTGCTGCCTATGATCCCAAATGGGGGC
RDE1a	TTGTTGGATATCATCGTCCCACACCAGGAGCATGTGCTGCCTATGATCCCAAATGGGGGC
RDA4	TTGTTGGATATCATCGTCCCACACCAGGAGCATGTGCTGCCTATGATCCCAAGTGGGGGC
STEa	TTGTTGGATATCATCGTCCCACACCAGGAGCATGTGCTGCCTATGATCCCAAGTGGGGGC
SDA2a	TTGTTGGATATCATCGTCCCACACCAGGAGCATGTGCTGCCTATGATCCCAAATGGGGGC
SDA1a	TTGTTGGATATCATCGTCCCACACCAGGAGCATGTGCTGCCTATGATCCCAAGTGGGGGC

Supplementary Figure S1b. Sequence alignment of a region of the *ALS* gene. Red box indicates the resistance-conferring *ALS* mutations from G to A at nucleotide position 1880, causing non-synonymous mutation from serine (AGT) to asparagine (AAT).

```
RBC2    TTGTTGGATATCATCGTCCCACACCAGGAGCATGTGCTGCCTATGATCCCAAATGGGGGC
RAB2    TTGTTGGATATCATCGTCCCACACCAGGAGCATGTGCTGCCTATGATCCCAAATGGGGGC
RKK     TTGTTGGATATCATCGTCCCACACCAGGAGCATGTGCTGCCTATGATCCCAAATGGGGGC
RKJ     TTGTTGGATATCATCGTCCCACACCAGGAGCATGTGCTGCCTATGATCCCAAATGGGGGC
RKH     TTGTTGGATATCATCGTCCCACACCAGGAGCATGTGCTGCCTATGATCCCAAATGGGGGC
RKL     TTGTTGGATATCATCGTCCCACACCAGGAGCATGTGCTGCCTATGATCCCAAATGGGGGC
RKI2    TTGTTGGATATCATCGTCCCACACCAGGAGCATGTGCTGCCTATGATCCCAAGTGGGGGC
RKG     TTGTTGGATATCATCGTCCCACACCAGGAGCATGTGCTGCCTATGATCCCAAATGGGGGC
RKI1    TTGTTGGATATCATCGTCCCACACCAGGAGCATGTGCTGCCTATGATCCCAAATGGGGGC
RBB1    TTGTTGGATATCATCGTCCCACACCAGGAGCATGTGCTGCCTATGATCCCAAATGGGGGC
RKE     TTGTTGGATATCATCGTCCCACACCAGGAGCATGTGCTGCCTATGATCCCAAATGGGGGC
SPC5    TTGTTGGATATCATCGTCCCACACCAGGAGCATGTGCTGCCTATGATCCCAAGTGGGGGC
STB     TTGTTGGATATCATCGTCCCACACCAGGAGCATGTGCTGCCTATGATCCCAAGTGGGGGC
STBa    TTGTTGGATATCATCGTCCCACACCAGGAGCATGTGCTGCCTATGATCCCAAGTGGGGGC
SKA     TTGTTGGATATCATCGTCCCACACCAGGAGCATGTGCTGCCTATGATCCCAAGTGGGGGC
SDA3    TTGTTGGATATCATCGTCCCACACCAGGAGCATGTGCTGCCTATGATCCCAAGTGGGGGC
SCD     TTGTTGGATATCATCGTCCCACACCAGGAGCATGTGCTGCCTATGATCCCAAGTGGGGGC
SPA2    TTGTTGGATATCATCGTCCCACACCAGGAGCATGTGCTGCCTATGATCCCAAGTGGGGGC
SPC4    TTGTTGGATATCATCGTCCCACACCAGGAGCATGTGCTGCCTATGATCCCAAATGGGGGC
SAB1    TTGTTGGATATCATCGTCCCACACCAGGAGCATGTGCTGCCTATGATCCCAAGTGGGGGC
SPC3    TTGTTGGATATCATCGTCCCACACCAGGAGCATGTGCTGCCTATGATCCCAAATGGGGGC
SDA1    TTGTTGGATATCATCGTCCCACACCAGGAGCATGTGCTGCCTATGATCCCAAGTGGGGGC
STE     TTGTTGGATATCATCGTCCCACACCAGGAGCATGTGCTGCCTATGATCCCAAGTGGGGGC
SKB     TTGTTGGATATCATCGTCCCACACCAGGAGCATGTGCTGCCTATGATCCCAAGTGGGGGC
SDA2    TTGTTGGATATCATCGTCCCACACCAGGAGCATGTGCTGCCTATGATCCCAAGTGGGGGC
STC     TTGTTGGATATCATCGTCCCACACCAGGAGCATGTGCTGCCTATGATCCCAAGTGGGGGC
SPC2    TTGTTGGATATCATCGTCCCACACCAGGAGCATGTGCTGCCTATGATCCCAAATGGGGGC
SPB1    TTGTTGGATATCATCGTCCCACACCAGGAGCATGTGCTGCCTATGATCCCAAATGGGGGC
SPC1    TTGTTGGATATCATCGTCCCACACCAGGAGCATGTGCTGCCTATGATCCCAAGTGGGGGC
```

Supplementary Figure S1b. Cont. Sequence alignment of a region of the *ALS* gene. Red box indicates the resistance-conferring *ALS* mutations from G to A at nucleotide position 1880, causing non-synonymous mutation from serine (AGT) to asparagine (AAT).

Ref-Indica	GCATTCAAGGACATGATCCTGGATGGTGATGGCAGGACTGTGTATTAATCTATAATCTGT
Ref-Japonica	GCATTCAAGGACATGATCCTGGATGGTGATGGCAGGACTGTGTATTAATCTATAATCTGT
Ref-US_HR	GCATTCAAGGACATGATCCCGGATGGTGATGGCAGGACTGTGTATTAATCTATAATCTGT
SKCL2	GCATTCAAGGACATGATCCTGGATGGTGATGGCAGGACTATGTATTAATCTATAATCTGT
RBB3a	GCATTCAAGGACATGATCCTGGATGGTGATGGCAGGACTATGTATTAATCTATAATCTGT
RPA4	GCATTCAAGGACATGATCCTGGATGGTGATGGCAGGACTATGTATTAATCTATAATCTGT
RDB1	GCATTCAAGGACATGATCCTGGATGGTGATGGCAGGACTATGTATTAATCTATAATCTGT
RAC2	GCATTCAAGGACATGATCCTGGATGGTGATGGCAGGACTATGTATTAATCTATAATCTGT
RBA1	GCATTCAAGGACATGATCCTGGATGGTGATGGCAGGACTATGTATTAATCTATAATCTGT
RBA2a	GCATTCAAGGACATGATCCTGGATGGTGATGGCAGGACTATGTATTAATCTATAATCTGT
RPB3	GCATTCAAGGACATGATCCTGGATGGTGATGGCAGGACTATGTATTAATCTATAATCTGT
RDA6a	GCATTCAAGGACATGATCCTGGATGGTGATGGCAGGACTATGTATTAATCTATAATCTGT
RAD3a	GCATTCAAGGACATGATCCTGGATGGTGATGGCAGGACTATGTATTAATCTATAATCTGT
RKEa	GCATTCAAGGACATGATCCTGGATGGTGATGGCAGGACTATGTATTAATCTATAATCTGT
RKC	GCATTCAAGGACATGATCCTGGATGGTGATGGCAGGACTATGTATTAATCTATAATCTGT
RDD2	GCATTCAAGGACATGATCCTGGATGGTGATGGCAGGACTATGTATTAATCTATAATCTGT
RBC3	GCATTCAAGGACATGATCCTGGATGGTGATGGCAGGACTATGTATTAATCTATAATCTGT
RSK2	GCATTCAAGGACATGATCCTGGATGGTGATGGCAGGACTATGTATTAATCTATAATCTGT
RDD1	GCATTCAAGGACATGATCCTGGATGGTGATGGCAGGACTATGTATTAATCTATAATCTGT
RCE	GCATTCAAGGACATGATCCTGGATGGTGATGGCAGGACTATGTATTAATCTATAATCTGT
RDE1	GCATTCAAGGACATGATCCTGGATGGTGATGGCAGGACTATGTATTAATCTATAATCTGT
RDC1	GCATTCAAGGACATGATCCTGGATGGTGATGGCAGGACTATGTATTAATCTATAATCTGT
RBB1a	GCATTCAAGGACATGATCCTGGATGGTGATGGCAGGACTATGTATTAATCTATAATCTGT
RAD1a	GCATTCAAGGACATGATCCTGGATGGTGATGGCAGGACTATGTATTAATCTATAATCTGT
RSK6	GCATTCAAGGACATGATCCTGGATGGTGATGGCAGGACTATGTATTAATCTATAATCTGT
RBA2	GCATTCAAGGACATGATCCTGGATGGTGATGGCAGGACTATGTATTAATCTATAATCTGT
RDA4a	GCATTCAAGGACATGATCCTGGATGGTGATGGCAGGACTGTGTATTAATCTATAATCTGT
RSK9	GCATTCAAGGACATGATCCTGGATGGTGATGGCAGGACTGTGTATTAATCTATAATCTGT
RTAa	GCATTCAAGGACATGATCCTGGATGGTGATGGCAGGACTGTGTATTAATCTATAATCTGT
RDE2a	GCATTCAAGGACATGATCCTGGATGGTGATGGCAGGACTGTGTATTAATCTATAATCTGT
RKD	GCATTCAAGGACATGATCCTGGATGGTGATGGCAGGACTATGTATTAATCTATAATCTGT
RBB2a	GCATTCAAGGACATGATCCTGGATGGTGATGGCAGGACTATGTATTAATCTATAATCTGT
RDA6	GCATTCAAGGACATGATCCTGGATGGTGATGGCAGGACTGTGTATTAATCTATAATCTGT
RAA1	GCATTCAAGGACATGATCCTGGATGGTGATGGCAGGACTATGTATTAATCTATAATCTGT
RDA5	GCATTCAAGGACATGATCCTGGATGGTGATGGCAGGACTATGTATTAATCTATAATCTGT
RKF	GCATTCAAGGACATGATCCTGGATGGTGATGGCAGGACTATGTATTAATCTATAATCTGT
RTA	GCATTCAAGGACATGATCCTGGATGGTGATGGCAGGACTGTGTATTAATCTATAATCTGT
RBB3	GCATTCAAGGACATGATCCTGGATGGTGATGGCAGGACTATGTATTAATCTATAATCTGT
RDA5a	GCATTCAAGGACATGATCCTGGATGGTGATGGCAGGACTGTGTATTAATCTATAATCTGT
RSK10	GCATTCAAGGACATGATCCTGGATGGTGATGGCAGGACTATGTATTAATCTATAATCTGT
RDC2	GCATTCAAGGACATGATCCTGGATGGTGATGGCAGGACTATGTATTAATCTATAATCTGT
RAD2	GCATTCAAGGACATGATCCTGGATGGTGATGGCAGGACTATGTATTAATCTATAATCTGT
RAD3	GCATTCAAGGACATGATCCTGGATGGTGATGGCAGGACTATGTATTAATCTATAATCTGT
RSK8	GCATTCAAGGACATGATCCTGGATGGTGATGGCAGGACTATGTATTAATCTATAATCTGT
RKFa	GCATTCAAGGACATGATCCTGGATGGTGATGGCAGGACTATGTATTAATCTATAATCTGT
RAD1	GCATTCAAGGACATGATCCTGGATGGTGATGGCAGGACTATGTATTAATCTATAATCTGT
RDE1a	GCATTCAAGGACATGATCCTGGATGGTGATGGCAGGACTATGTATTAATCTATAATCTGT
RDA4	GCATTCAAGGACATGATCCTGGATGGTGATGGCAGGACTGTGTATTAATCTATAATCTGT
STEa	GCATTCAAGGACATGATCCTGGATGGTGATGGCAGGACTGTGTATTAATCTATAATCTGT
SDA2a	GCATTCAAGGACATGATCCTGGATGGTGATGGCAGGACTATGTATTAATCTATAATCTGT
SDA1a	GCATTCAAGGACATGATCCTGGATGGTGATGGCAGGACTGTGTATTAATCTATAATCTGT
RBC2	GCATTCAAGGACATGATCCTGGATGGTGATGGCAGGACTATGTATTAATCTATAATCTGT
RAB2	GCATTCAAGGACATGATCCTGGATGGTGATGGCAGGACTATGTATTAATCTATAATCTGT
RKK	GCATTCAAGGACATGATCCTGGATGGTGATGGCAGGACTATGTATTAATCTATAATCTGT
RKJ	GCATTCAAGGACATGATCCTGGATGGTGATGGCAGGACTATGTATTAATCTATAATCTGT

Supplementary Figure S1c. Sequence alignment of a region of the *ALS* gene. Red box indicates the resistance-conferring *ALS* mutations from G to A at nucleotide position 1927, causing non-synonymous mutation from valine (GTG) to methionine (ATG).

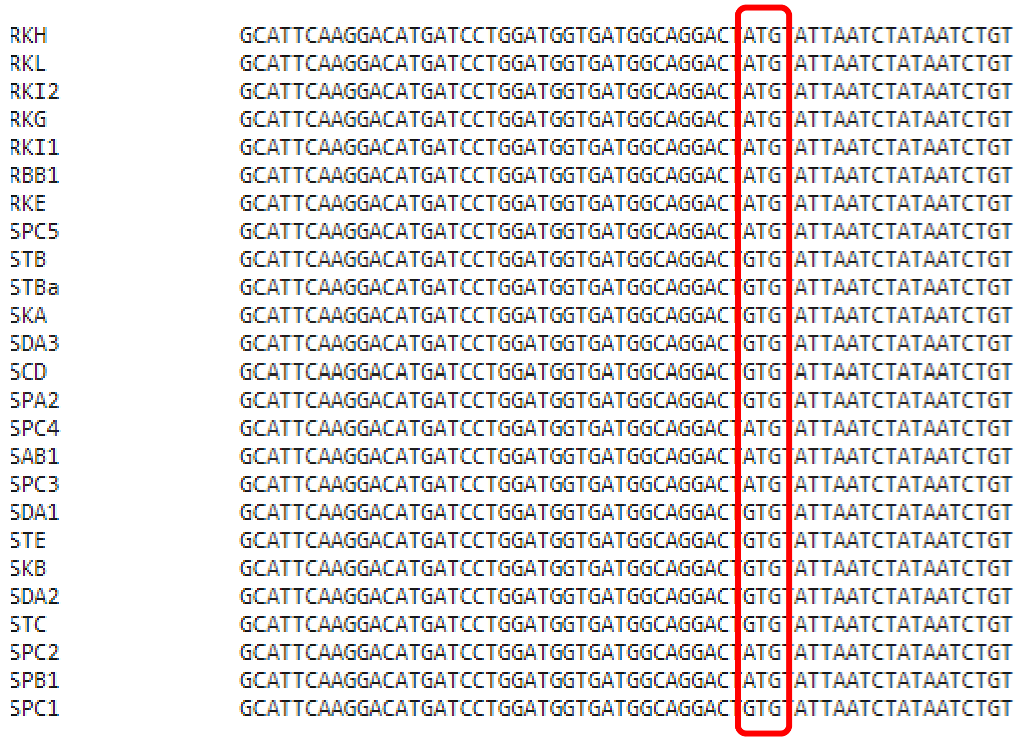

Supplementary Figure S1c. Cont. Sequence alignment of a region of the *ALS* gene. Red box indicates the resistance-conferring *ALS* mutations from G to A at nucleotide position 1927, causing non-synonymous mutation from valine (GTG) to methionine (ATG).

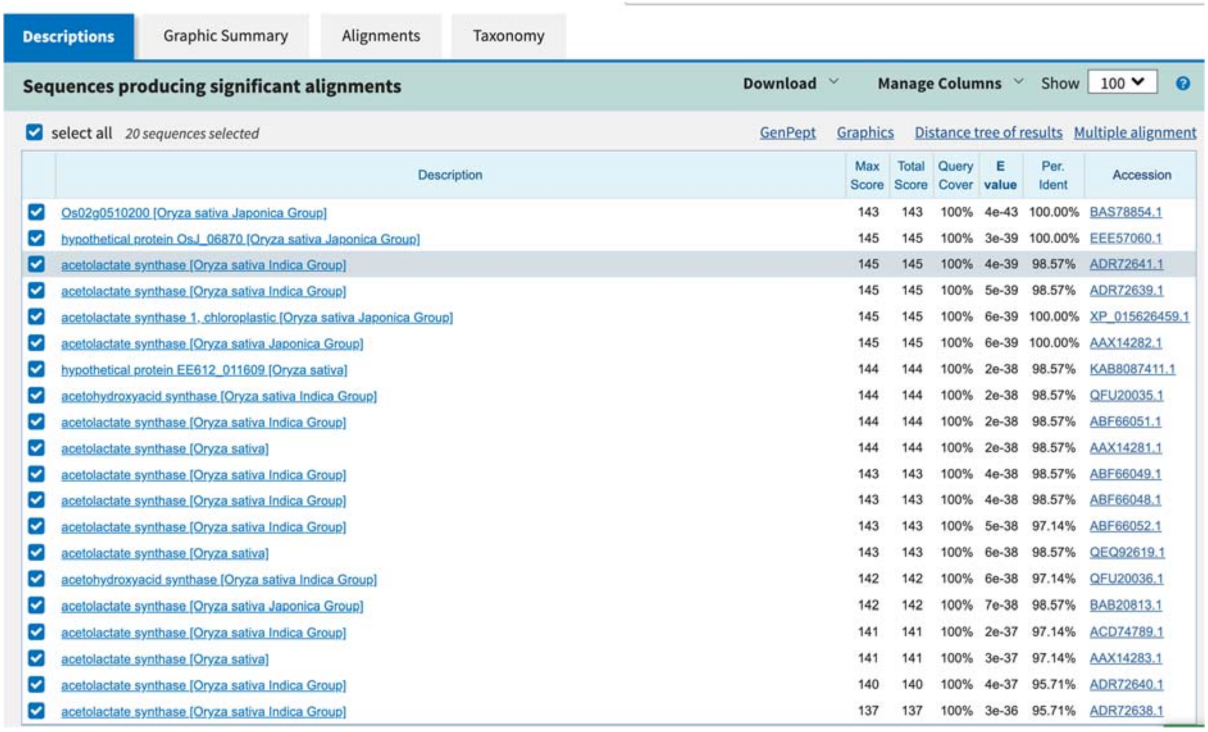

Descriptions | Graphic Summary | Alignments | Taxonomy

Sequences producing significant alignments Download Manage Columns Show 100

select all *20 sequences selected* GenPept Graphics Distance tree of results Multiple alignment

Description	Max Score	Total Score	Query Cover	E value	Per. Ident	Accession
Os02g0510200 [Oryza sativa Japonica Group]	143	143	100%	4e-43	100.00%	BAS78854.1
hypothetical protein OsJ_06870 [Oryza sativa Japonica Group]	145	145	100%	3e-39	100.00%	EEE57060.1
acetolactate synthase [Oryza sativa Indica Group]	145	145	100%	4e-39	98.57%	ADR72641.1
acetolactate synthase [Oryza sativa Indica Group]	145	145	100%	5e-39	98.57%	ADR72639.1
acetolactate synthase 1, chloroplastic [Oryza sativa Japonica Group]	145	145	100%	6e-39	100.00%	XP_015626459.1
acetolactate synthase [Oryza sativa Japonica Group]	145	145	100%	6e-39	100.00%	AAX14282.1
hypothetical protein EE612_011609 [Oryza sativa]	144	144	100%	2e-38	98.57%	KAB8087411.1
acetohydroxyacid synthase [Oryza sativa Indica Group]	144	144	100%	2e-38	98.57%	QFU20035.1
acetolactate synthase [Oryza sativa Indica Group]	144	144	100%	2e-38	98.57%	ABF66051.1
acetolactate synthase [Oryza sativa]	144	144	100%	2e-38	98.57%	AAX14281.1
acetolactate synthase [Oryza sativa Indica Group]	143	143	100%	4e-38	98.57%	ABF66049.1
acetolactate synthase [Oryza sativa Indica Group]	143	143	100%	4e-38	98.57%	ABF66048.1
acetolactate synthase [Oryza sativa Indica Group]	143	143	100%	5e-38	97.14%	ABF66052.1
acetolactate synthase [Oryza sativa]	143	143	100%	6e-38	98.57%	QEQ92619.1
acetohydroxyacid synthase [Oryza sativa Indica Group]	142	142	100%	6e-38	97.14%	QFU20036.1
acetolactate synthase [Oryza sativa Japonica Group]	142	142	100%	7e-38	98.57%	BAB20813.1
acetolactate synthase [Oryza sativa Indica Group]	141	141	100%	2e-37	97.14%	ACD74789.1
acetolactate synthase [Oryza sativa]	141	141	100%	3e-37	97.14%	AAX14283.1
acetolactate synthase [Oryza sativa Indica Group]	140	140	100%	4e-37	95.71%	ADR72640.1
acetolactate synthase [Oryza sativa Indica Group]	137	137	100%	3e-36	95.71%	ADR72638.1

Supplementary Figure S2. BLAST result for the acetolactate synthase (*ALS*) gene position in Chromosome 2 of the weedy rice (*Oryza sativa*).
